# Exploring parameter optimisation in machine learning algorithms for locomotor task discrimination using wearable sensors

**DOI:** 10.1038/s41598-025-17361-y

**Published:** 2025-10-09

**Authors:** L. D. Hughes, M. Bencsik, M. Bisele, C. T. Barnett

**Affiliations:** 1 School of Science and Technology, Nottingham Trent University, Clifton Lane, Nottingham, NG11 8NS UK; 2 University Hospitals of Coventry and Warwickshire NHS Trust, Coventry, UK; 3 Heidelberg University Hospital, Heidelberg, Germany

**Keywords:** Machine learning, Wearable sensors, Optimisation, Locomotion, Principal component analysis, Discriminant function analysis, Bone quality and biomechanics, Characterization and analytical techniques, Design, synthesis and processing, Biomarkers, Computer science

## Abstract

The accurate identification of locomotion states from wearable sensor data using machine learning relies heavily on carefully selecting algorithm parameters, which remains a challenging task. This study systematically optimised key parameters—including window length, sampling frequency, temporal resolution, overlapping value, and normalisation effects—to enhance the accuracy of machine learning models for distinguishing different locomotor tasks. Our study was conducted on participants (*N* = 35, 19 ♂10 ♀, 27.4 ± 26.5 years, 1.74 ± 0.8 m, 71.5 ± 11.3 kg) who wore accelerometers on the sacrum, thighs and shanks. Principal component and discriminant function analyses were applied to acceleration data from three locomotor tasks: self-selected slow, normal and fast walking. The parameters explored for the optimisation of the algorithm were accelerometer window length, sampling frequency, spectral temporal resolution, overlapping value, and accelerometer amplitude normalisation effects. Unnormalised data, with longer feature window lengths and decreasing temporal resolutions, yielded the highest quality discrimination. Setting the sampling rate to 40 Hz and overlapping value to 66% provided optimal discrimination. Baseline results highlight that the sacrum is the best-performing location, yet optimal (longer) window lengths, and optimal (shorter) temporal resolutions change the best-performing sensor attachment location to the shanks. Specific values of parameters were found to be optimal for our study, and these results can guide manufacturers, engineers, and researchers in designing wearable devices and machine learning algorithms that more effectively identify locomotor tasks. Practitioners and clinicians may also use these findings to select appropriate tools or methodologies tailored to their specific research or clinical objectives.

## Introduction

In gait recognition, machine learning is applied to transform raw data sets into models that aim to identify a particular activity. In the context of human activity recognition, wearable sensors are found in several forms, e.g., inertial measurement units (IMUs)^1–3^ collecting raw accelerations and gyroscopic rotations^4–6^, IMUs built into smartphones^7–9^ typically collect and store the raw data for the machine learning application. Machine learning algorithms provide the ability to cluster movement data into different categories, e.g. walking, running, changing terrain, and climbing stairs^2,3,10–12^, facilitating the collection of ecologically valid information from participants. Knowing what activity individuals perform from data collected by a wearable sensor has exciting potential clinical benefits. For example, deviations in locomotion and activities are known predictors of specific pathologies such as Parkinson’s disease^13,14^, cerebral palsy^15^ and multiple sclerosis^16^. Detailed information on locomotion has the potential to aid prosthetic and orthotic prescriptions^17^. However, choosing the optimal parameters for a given wearable sensor located at a particular part of the body to discriminate specific locomotor tasks is an issue. A machine learning algorithm’s ability to discriminate or classify data to determine locomotor activities depends on numerous parameters. Those explored in the current study affect the feature or the key characteristics extracted from the raw measurement. In the case of using a 2-dimensional Fourier Transform (2D-FT)^18^, this includes the sampling frequency - how often samples are taken from the sensor, the window length - how long the data is for one feature computation, the overlapping value - how often the Fast Fourier Transform (FFT) windows overlap, and the temporal resolution - the time duration of the data used for one spectrum computation.

Previously reported accelerometer sampling frequencies for activity recognition typically range from 20 Hz^19^ to 100 Hz^12,20^, with reported values also occasionally surpassing 500 Hz^10,11^. The Nyquist theorem provides a relationship between the sampling rate and the maximum frequency content of the continuous time signal it represents. It stipulates that the sampling rate should be at least twice as large as the one of interest in the time signal. Choosing an appropriate sampling frequency is a delicate balance; too low and crucial information could be missed; too high, and there is a risk of overfitting the data, providing features with irrelevant information. Higher sampling frequencies consume more battery power in sensors, negating the ability to collect data for a long time, so lower frequencies that can still collect discriminating signal features are ideal.

**Fig. 1:**
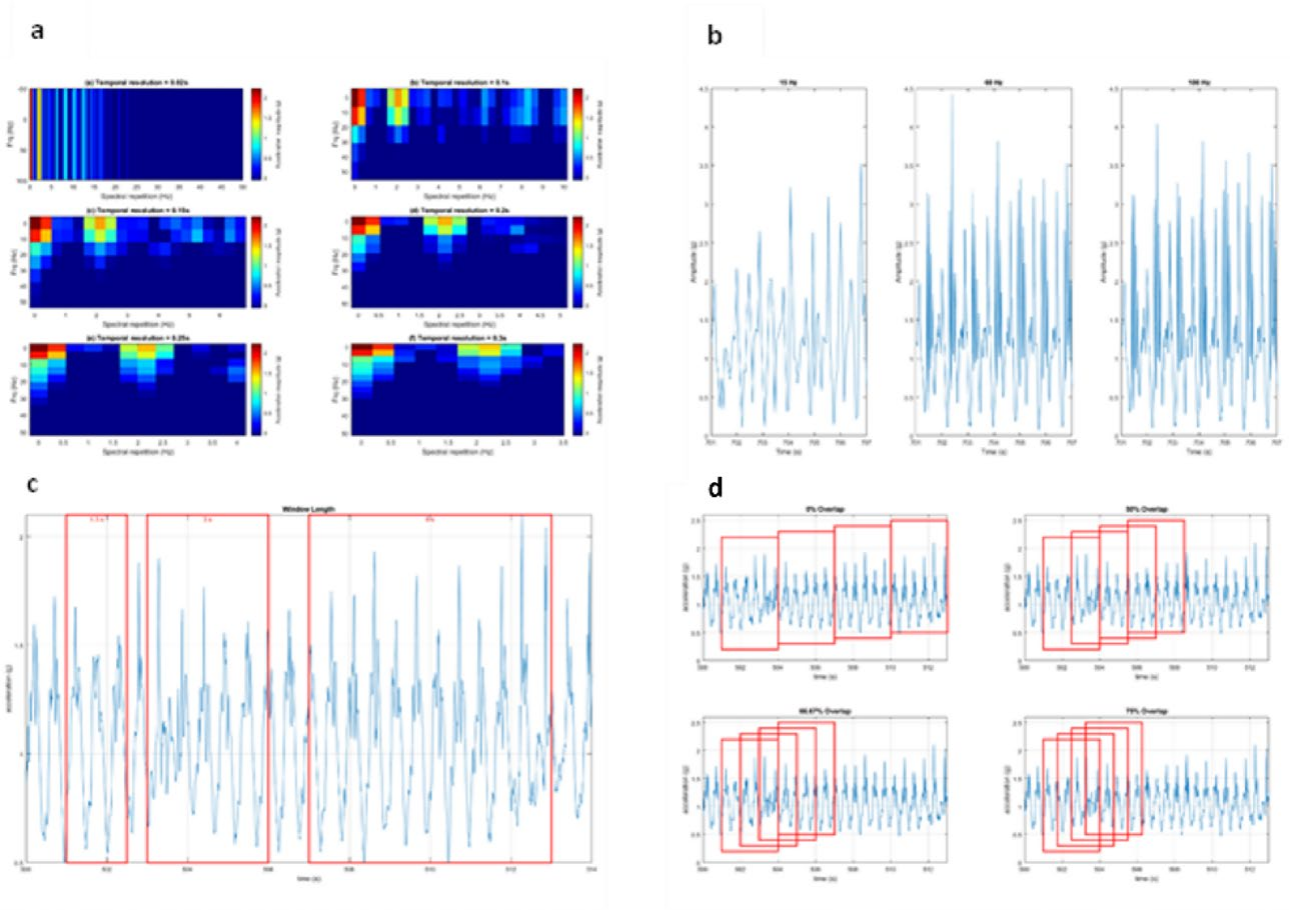
(**a**) An example displaying the effects on the 2DFT of the spectrum’s temporal resolution (0.02–0.3 s). Lower resolutions capture more detail in the spectral repetitions; higher resolutions capture detail in the spectrum repeating itself. (**b**) Highlights the effects of lower and higher sampling frequencies on the signal (15 Hz, 60 Hz, and 100 Hz). Lower frequencies risk missing essential signal features. (**c**) Visually showcases different lengths of window, 1 s, 3s, and 6 s. (**d**) Shows four differing overlapping window values on the signal (0% − 75%). Higher overlapping values result in windows sharing more of the same information from a signal. The number of points along a vertical line (power spectrum) is one half of the product of the sampling rate by the temporal resolution.

Feature selection is crucial when building any machine-learning algorithm to discriminate between locomotor activities using wearable sensors; even the best-performing machine-learning algorithm will underperform if provided with a poorly chosen feature. A key process in feature selection is the transformation of a signal. Transforming signals commonly involves numerical methods like the Fourier Transform^21^ to dissect waveforms into their sine wave components. A Fast Fourier Transform facilitates rapid decomposition, deconstructing waveforms into sinusoidal components^22,23^. The 2-dimensional Fourier Transform (2D-FT) takes this a step further by performing a dual spectral computation with FFT undertaken across both the vertical (amplitude) and horizontal (time) axes, revealing the frequency of spectral repetitions throughout the signal’s length. This transformation is most useful when dealing with complex signals that repeat themselves, such as in the case of 2DFT Nuclear Magnetic Resonance^24^, or such for a short section of gait data. When generating a 2D-FT, choosing the appropriate temporal resolution is vital, as it determines the timeframe over which each spectrum is analysed, significantly influencing the level of detail captured. A higher temporal resolution allows for the capture of finer vertical spectral details over longer periods, deteriorating the resolution of the spectral repetition analysis. Conversely, a lower temporal resolution improves the resolution of the spectral repetition analysis, at the expense of a less detailed visualisation of the spectrum which is repeating itself (Fig. 1). Selecting an excessively high temporal resolution may prioritise capturing fine-grained vertical spectral details but fail to adequately represent the broader, repetitive patterns of locomotor activities critical for discrimination. On the other hand, an overly low temporal resolution enhances the clarity of spectral repetition but risks oversimplifying or losing essential details about the signal’s variability.

To effectively analyse the accelerometer signal data, features must be analysed from a known time window in the data when a particular action is taking place. The ideal window size balance includes enough detail and avoids two separate activities overlapping in the same data slab. Shorter windows excel at identifying quick variations in a signal, while longer windows provide a more accurate perspective on the signal’s trends over time. Previous research in activity recognition using accelerometers has used varying lengths, with suggestions of optimal window lengths ranging from 1.6 seconds (5) to 15 seconds^25^. Previous investigations into window size have suggested that lengths in the region of 3–7 seconds yield the best classification accuracies^26^.

Implementing a fixed-size sliding window is the most prevalent method for windowing data in activity recognition tools that utilise wearable sensors^26^. This approach involves overlapping windows to ensure no gaps between them, allowing for more frequent analysis than the number of samples taken. For example, a 50% overlapping window ensures half of the signal is shared between two consecutive windows (Fig. 1)^26^. In activity recognition studies utilising wearable sensors, the sliding window approach reports overlapping of 20–75%^27^. Research has suggested that larger overlaps yield better classification results^26^. Although increased overlap may lead to improved outcomes, it also necessitates additional computing time and memory. This added computational burden can significantly impact battery life in wearable devices, potentially limiting their usability for extended periods or in resource-constrained applications such as long-term monitoring or remote health assessments.

When building a machine learning algorithm, users can normalise the amplitude of the feature or not. Normalisation is a preprocessing step that provides a method of adjusting the magnitude of the data into a standard scale without distorting the range of the signal values. Not every study opts to normalise their data, with approximately 61% of articles on the automatic recognition of gait patterns using machine learning choosing to do so^28^. When utilising machine learning to identify activities with accelerometers, the effects of normalisation remain relatively unexplored. Normalisation can provide a more robust classification^28^, potentially enhancing the interpretability of the data; however, altering the raw signal could result in the algorithm having fewer distinctive frequencies available for clustering, as, e.g. faster gait speeds often results in higher magnitude acceleration.

The location of wearable sensors is a critical consideration in gait analysis, as it influences the quality of the acceleration signals and, consequently, the parameters needed for effective feature extraction and classification. Common attachment sites, such as the lower back, thighs, and shanks, have been shown to successfully capture gait dynamics due to their proximity to the centre of mass, or for producing large acceleration signals^18^. While previous studies have explored optimal sensor locations for discriminating cyclical locomotor tasks, the current study focuses on the optimisation of feature-based parameters, and whether the optimisation is location dependent.

Given the importance of selecting a sampling rate, window length, temporal resolution, overlapping value and whether to normalise the acceleration signals when using a 2DFT for feature extraction, the current study aimed to systematically explore their optimisation when discriminating three separate locomotor tasks, using a feature selection-based machine learning algorithm using wearable sensors attached at different body locations.

## Methods

### Participants

Participants (*N* = 35, 19 ♂10 ♀, 27.4 ± 26.5 years, 1.74 ± 0.8 m, 71.5 ± 11.3 kg) with no known injuries or illnesses at the time of testing were recruited. Participants signed written informed consent forms prior to participation. The study was conducted in accordance with the Declaration of Helsinki, and approved by the Institutional Human Ethics Committee of Nottingham Trent University (protocol code 595 28 October 2020).

### Experimental design

**Fig. 2:**
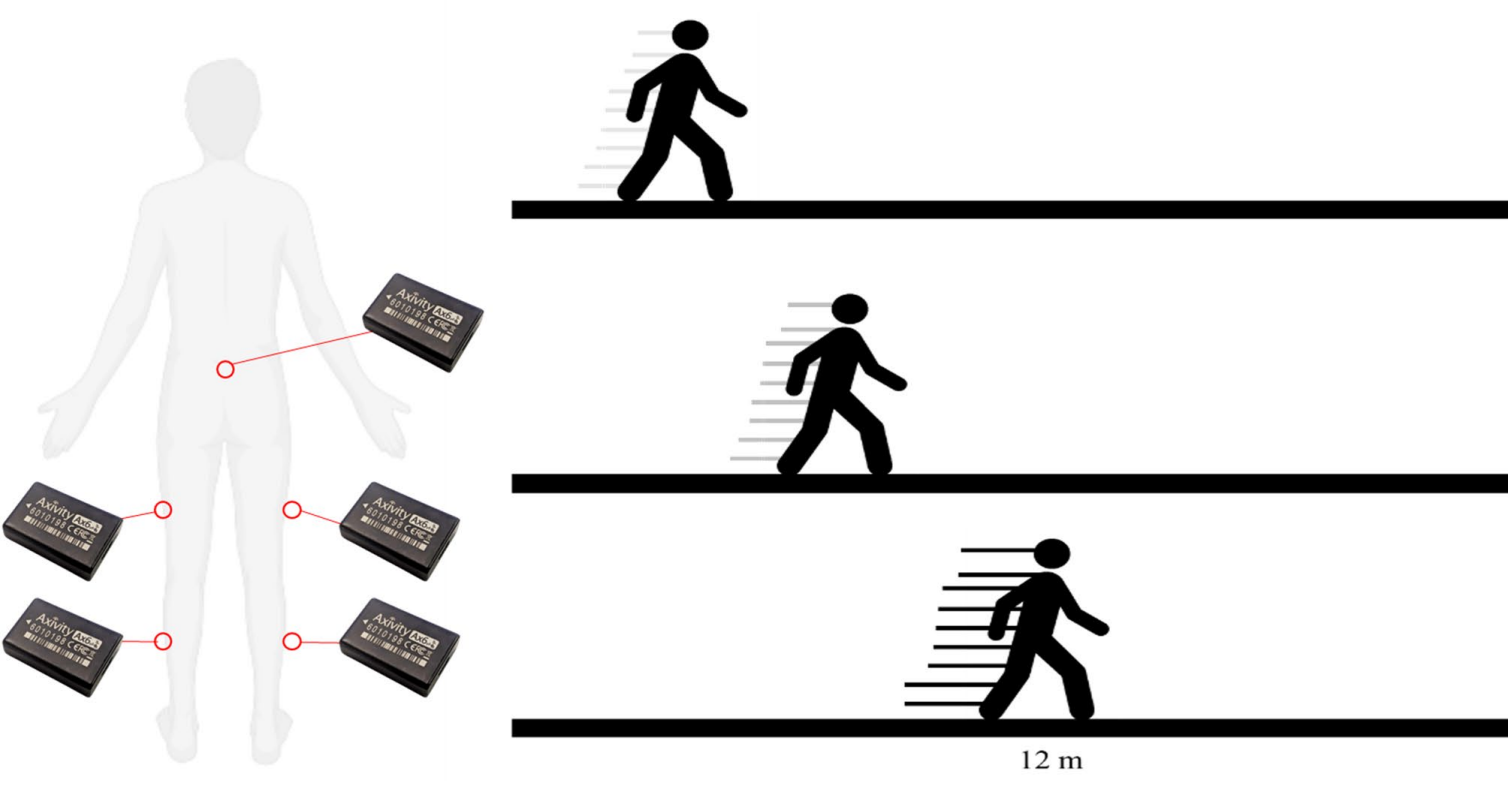
Five Axivity AX6 sensors were attached to the sacrum, the midpoint of the lateral thighs, and the midpoint of the lateral shank segments. Participants completed three experimental conditions: slow, normal, and fast walking. A total distance of 120 m was completed per condition on a 12 m walkway.

Participants arrived at the university wearing t-shirts, shorts or skin-tight leggings, and their own trainers. They completed three locomotion tasks along a 12-metre walkway (Fig. 2). Each condition required participants to walk at either a self-selected slow, normal or fast pace; under the guidance provided to all participants, “normal” had to be faster than the “slow” condition and “fast” had to be faster than the normal condition. The mean walking speeds for the slow, normal, and fast conditions were 0.8 m/s (± 0.22), 1.1 m/s (± 0.07), and 1.3 m/s (± 0.10), respectively. Participants walked a total of 120 m for each of the three walking conditions, and the order of experimental conditions was randomised between participants via a random number generator and periods of one-minute quiet standing separated conditions.

*Instrumentation* Triaxial accelerometer sensors (Axivity AX6, York, UK) sampled at 100 Hz and 8 g range were used. Sensors were attached to the skin with double-sided adhesive tape at the sacrum, the approximate midpoint of lateral left and right thigh segments, and the approximate midpoint of lateral left and right shank segments (Fig. 2). Sensors were configured to record accelerations in X, Y, and Z orthogonal planes. All accelerometer signals were visually inspected and remained within the ± 8 g range, as expected for walking activities.

### Pre-processing

The magnitude of the acceleration vector for the raw acceleration time course was used: $a=\sqrt{{a}_{x\:}^{2}+{a}_{y\:}^{2}+{a}_{z}^{2}}$

Linear interpolation allowed the synchronisation of each accelerometer to the same digital time stamp, as the sensors were found to present slight variations in sampling frequency, even though they had been pre-set to 100 Hz.

### Baseline machine learning algorithm

**Fig. 3:**
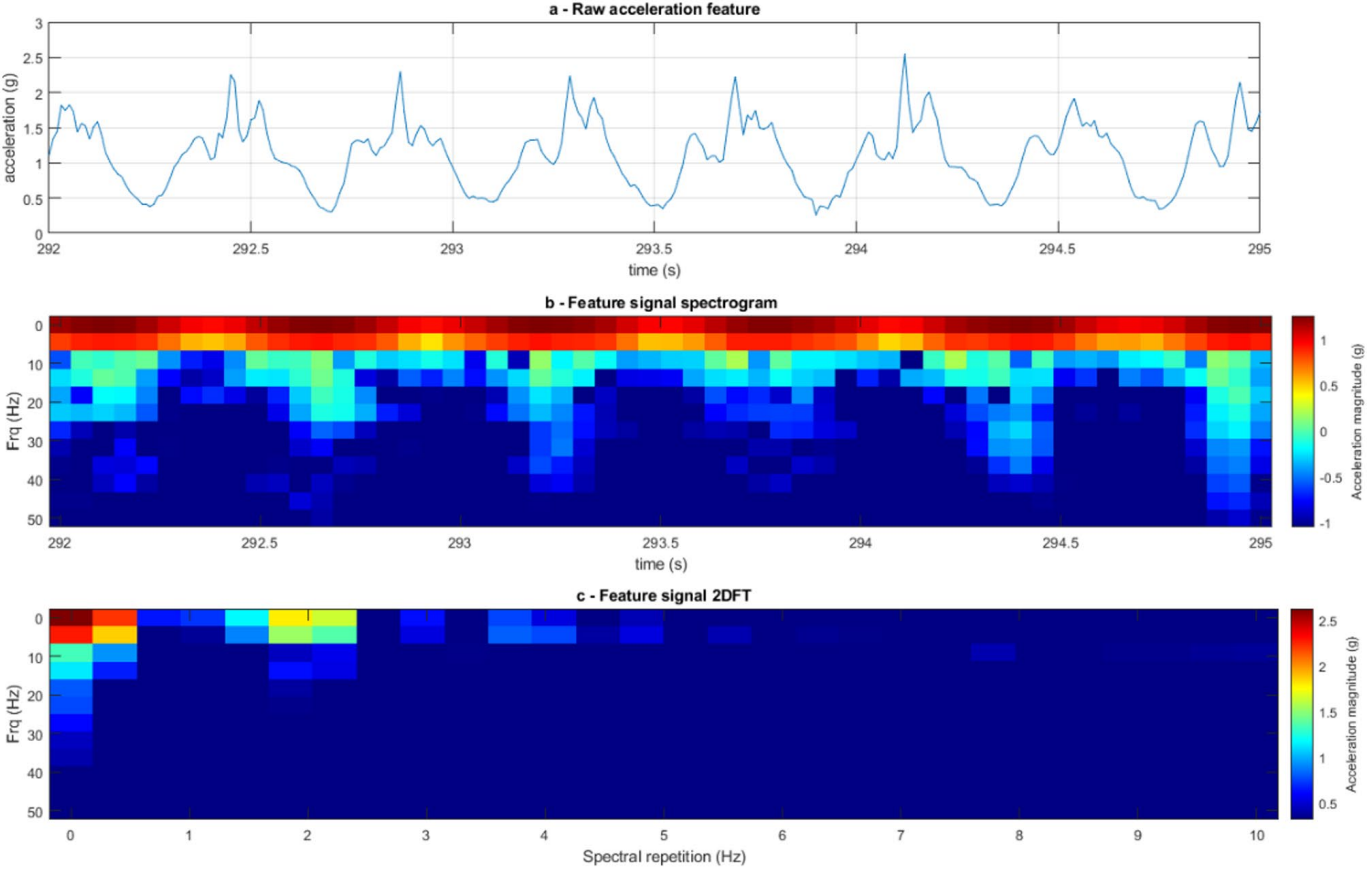
(**a**) Time course of the raw acceleration signal during normal walking, showcasing six peaks in acceleration amplitude across a period of three seconds. (**b**) A spectrogram of the three-second acceleration signal highlights six periods of large frequency bandwidth. (**c**) The Two-dimensional Fourier Transform image of the experimental condition highlights the spectrum of a repeated signal, just under twice per second.

Prior to exploring any optimisation, a baseline algorithm, consisting of starting parameters was executed, with parameter values selected from previous works^18^. Two-dimensional Fourier Transform analysis (2DFT)^23^ was implemented as the feature to analyse the raw acceleration signal for each experimental condition (Fig. 3). For the training of the machine learning, signal features with window lengths of three seconds were selected from periods of known gait category (slow, normal or fast). The temporal resolution, determining the time duration of data used to compute a single frequency spectrum, was set at 300 ms. A window overlap factor of 50% was applied to segment the time-domain data to enhance the detection of transient frequency characteristics during the 2DFT analysis. This resulted in adjacent segments sharing 50% of their data points.

**Fig. 4:**
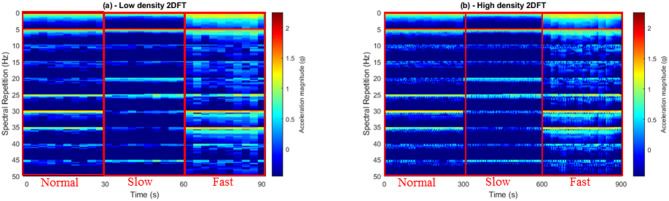
Two sets of Two-dimensional Fourier Transform images, each illustrating three experimental conditions related to walking. (**a**) A stacked series of stretched images demonstrates normal, slow, and fast walking conditions. For each condition, ten 3-second long spectra are stacked, with dark red and dark blue, respectively, indicating higher and lower acceleration magnitudes (**b**) A higher-density series captures the same experimental conditions but with a temporal scan increment of 0.1 s over one second, resulting in 100 × 3-second long spectra per condition. The rectangular selections highlight the normal, slow, and fast walking conditions in both sets of images.

The 2DFT was applied individually to each selected three-second long time window, generating features exclusively within a frequency domain representation, as described in previous work^18^. This approach ensured that the resulting feature remained consistent regardless of absolute timings, or phase of the specific analysis window. The 2DFT matrix was then stretched into an array format. Each 2DFT array was then stacked, creating a new, larger 2DFT matrix with ten stretched features for each walking condition (Fig. 4). These matrices visually revealed the groups with similar fundamental frequency and associated harmonics present in the acceleration signal for a given category of gait, allowing for the observation of repeated spectral patterns. The series of 2DFT was scrutinised via visual inspection, which was crucial to identify and exclude any instances of error, in particular when inadvertently selecting data segments containing signals in which participants were turning around the cones on the set-up walkway or periods of gait acceleration or deceleration.

Subsequently, a higher-density matrix of 2-dimensional Fourier transform features were also calculated. Building on the initial ten features per gait category, using 2DFT with time increments of 0.1 s, spanning a duration of one second, a new training database (TDB) was computed. This high-density scan yielded 100 features for each experimental condition. This resulted in a total of 300 2DFT spectra. The computation of both high and low-density 2DFT TDB serves a separate purpose. Firstly, the low-density scan provides a comprehensive overview of the frequency content within the data, allowing for the training of computer algorithms for the discrimination of different gaits. Secondly, the additional high-density matrix is a valuable tool for quantifying the extent of deviations exhibited by the features within a specific gait condition. The purpose of the high density database was to identify components that displayed variation and enable their clustering (Fig. 4).

A simple machine learning algorithm consisting of Principal Component Analysis (PCA) and Discriminant Function Analysis (DFA) was employed on each training database to enable the clustering of the gait modalities, followed by the quantification of the deviations around the centroid. The PCA was done by diagonalising the covariance matrix, as all input parameters consist of accelerometer data. Principal Component scores (a set of values that transform the dependent variable within a multivariate database into a reduced set of principal components^29^ were generated for each experimental condition. Principal Components for each variable were calculated to the 80_th_ percentile. This approach captured 80% of the total variance within each gait modality, participant and sensor, ensuring adaptability to the inherent variability within each dataset.

Discriminant Function Analysis (also known as Linear Discriminant Analysis) identified discriminating features between the gait modalities to cluster the reduced dimensionality data further, as required by the user. DFA is a supervised training technique optimising discrimination between predefined conditions^29^.

**Fig. 5:**
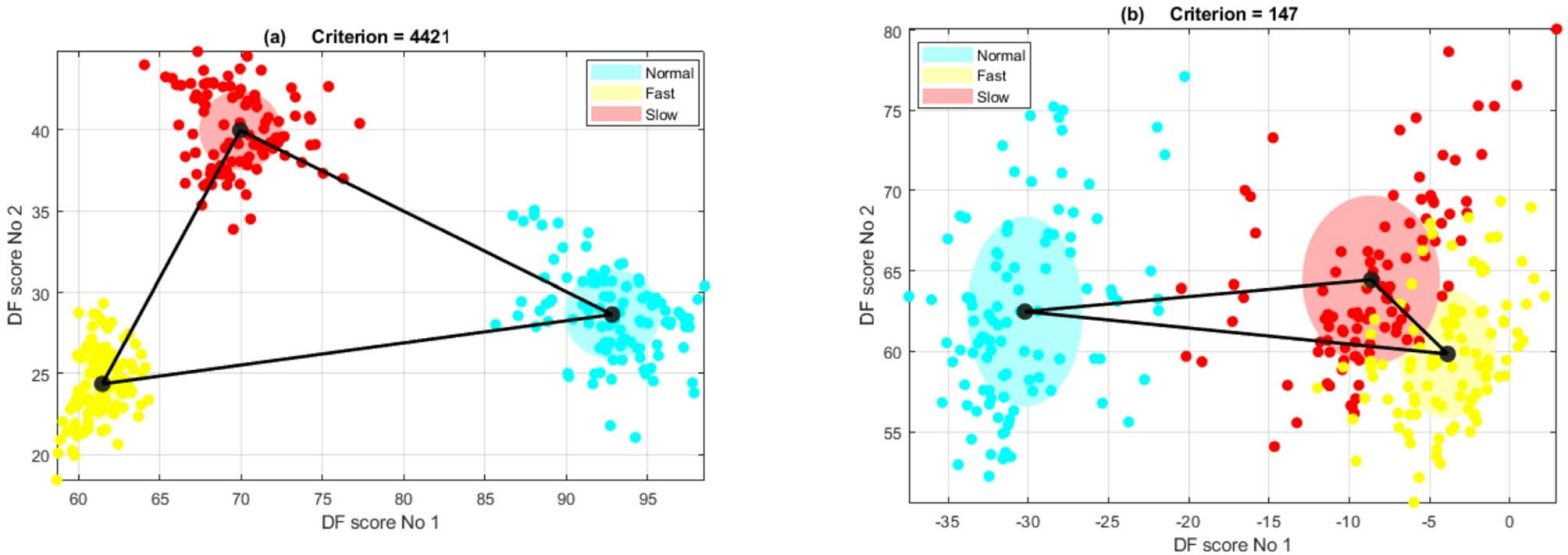
The discriminant function scores reveal distinct clustering of the three activities of interest. (**a**) Successful discrimination is evident with a criterion score exceeding 500. The cloud centroids display substantial separation, and the standard deviations within clusters are comparatively small. (**b**) Illustrates unsuccessful discrimination, marked by a criterion score below 500. In this case, two experimental conditions cloud centroids (normal and fast walking) are closely positioned, and the cluster standard deviations are relatively large.

After applying DFA within two dimensions, the discriminant function (DF) scores 1 and 2 were plotted (Fig. 5). A criterion score was then computed to assess the quality of discrimination between the three conditions. This score was derived from the ratio of the product of the three centroid distances between the three clusters to the product of the three standard deviations of each cluster, as demonstrated in our previous publication^18^. Strong discriminatory performance is indicated by simultaneously fulfilling a substantial distance between centroids and low standard deviations within each condition. The discrimination criterion was formulated as the following:

$$\text{Discrimination criterion} = \left( {\frac{{dis{t_1}*dis{t_2}*dis{t_3}}}{{scatte{r_{01}}*scatte{r_{02}}*scatte{r_{03}}}}} \right)$$

Where Dist_1 represents the distance between centroids of fast and normal clouds, Dist_2 is the distance between fast and slow centroid clouds, and Dist_3 is that of normal and slow cluster centroids. Scatter_01, Scatter_02 and Scatter_03 are calculated by determining the standard deviations of the distances of each data point to their centroid for each experimental condition (Fig. 5).

Experimenters visually examined the degree of overlap between the clouds corresponding to different gait types to subjectively relate, approximately, the success of discrimination between the three locomotion modes to the value of the discrimination criterion. When conditions severely overlapped, discrimination was considered unsuccessful. A discrimination criterion value exceeding a value around 500 most often indicated zero overlap between clouds for different walking conditions. Some overlap between conditions was observed when the criterion ratio was below 500. Therefore, a success criterion threshold value of 500 was chosen. This threshold was empirically derived and aligns with observed cluster separability across tasks. The outcomes of successful and unsuccessful discrimination attempts were documented for all five sensors. This comprehensive evaluation provided each sensor location with a meaningful count of successful discriminations achieved between the three experimental conditions.

### Optimisation process

Following the development of the baseline machine learning algorithm (comprising parameters set to a sampling rate of 100 Hz, window-length of 3 s, temporal resolution of 0.3 s and overlapping value of 50% that did not use normalisation), optimal parameters for discriminating gait modalities were systematically explored one by one. Each parameter had their values exhaustively swept one at a time, keeping all other ones constrained to that of the original baseline scan.

### Normalisation

The first step was to see the impact of normalising accelerometer signal amplitudes. Before applying any machine learning to the 2DFT matrices, the acceleration signal was normalised by dividing each measurement by the mean of the signal in the feature, meaning acceleration magnitude variability was removed, providing an initial comparison data set of normalised vs. not normalised signals.

### Temporal resolution

The temporal resolution was the first parameter to be scanned for the optimal value, between values of 20 and 300 ms, in incremented steps of 10 ms.

### Sampling frequency

The second parameter to be explored was the sampling frequency, between values of 25 and 100 Hz in incremented steps of 5 Hz. Rates lower than the original were obtained during post processing by interpolating the raw accelerometer data.

### Window length

The following parameter that was scanned was window length between 0.9 and 6 s values in increments of 0.3 s.

### Overlapping value

The final parameter to be explored was the overlapping values. Six overlapping values: 0%, 50%, 66%, 75%, 80%, and 88.34% were analysed.

## Results

### Baseline

**Fig. 6:**
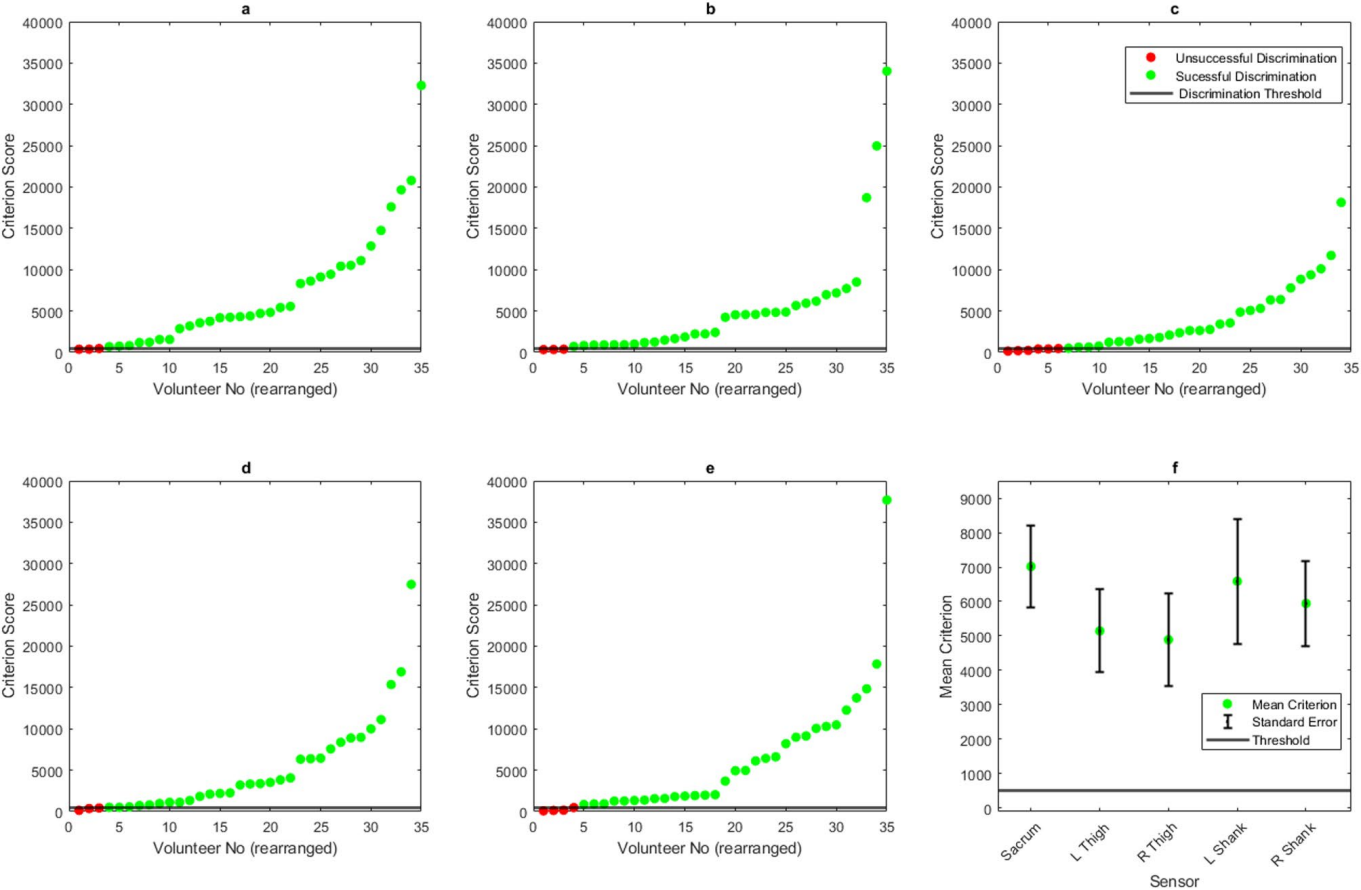
Baseline criterion values for all participants; values arranged from left-right, lowest to highest criterion for (**a**) sacrum, (**b**) left thigh, (**c**) right thigh, (**d**) left shank, (**e**) right shank sensors. (**f**) Displays the mean criterion scores for each sensor.

The baseline parameter scan results show that the algorithm successfully discriminated gait modes, with 91% of outcomes being successful in the sensor attached to the sacrum, and a mean criterion of 7009. The left and right thighs had a combined success rate of 87% with a combined mean criterion of 4943, and the left and right shanks had a success rate of 90%, with a mean criterion of 6212 (Fig. 6).

### Normalisation

**Fig. 7:**
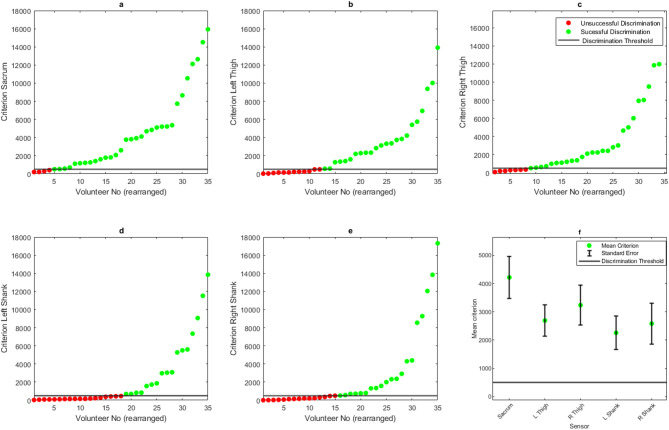
Criterion values for all participants following data normalisation; values arranged from left-right, lowest to highest criterion for (**a**) sacrum, (**b**) left thigh, (**c**) right thigh, (**d**) left shank, (**e**) right shank sensors. (**f**) Displays the mean criterion scores for each sensor.

Normalisation negatively affected the outcome of the algorithm. The sensor at the sacrum was successful for 88% of outcomes, the thighs 66% and the shanks 53%. The overall mean criterion value also dropped for each attachment location, from 7009 to 4216 in the sacrum, 5034 to 2692 and 4852 to 3237 in the left and right thighs, and 6531 to 2258 and 5901 to 2584 in the left and right shanks (Fig. 7).

### Temporal resolution

**Fig. 8:**
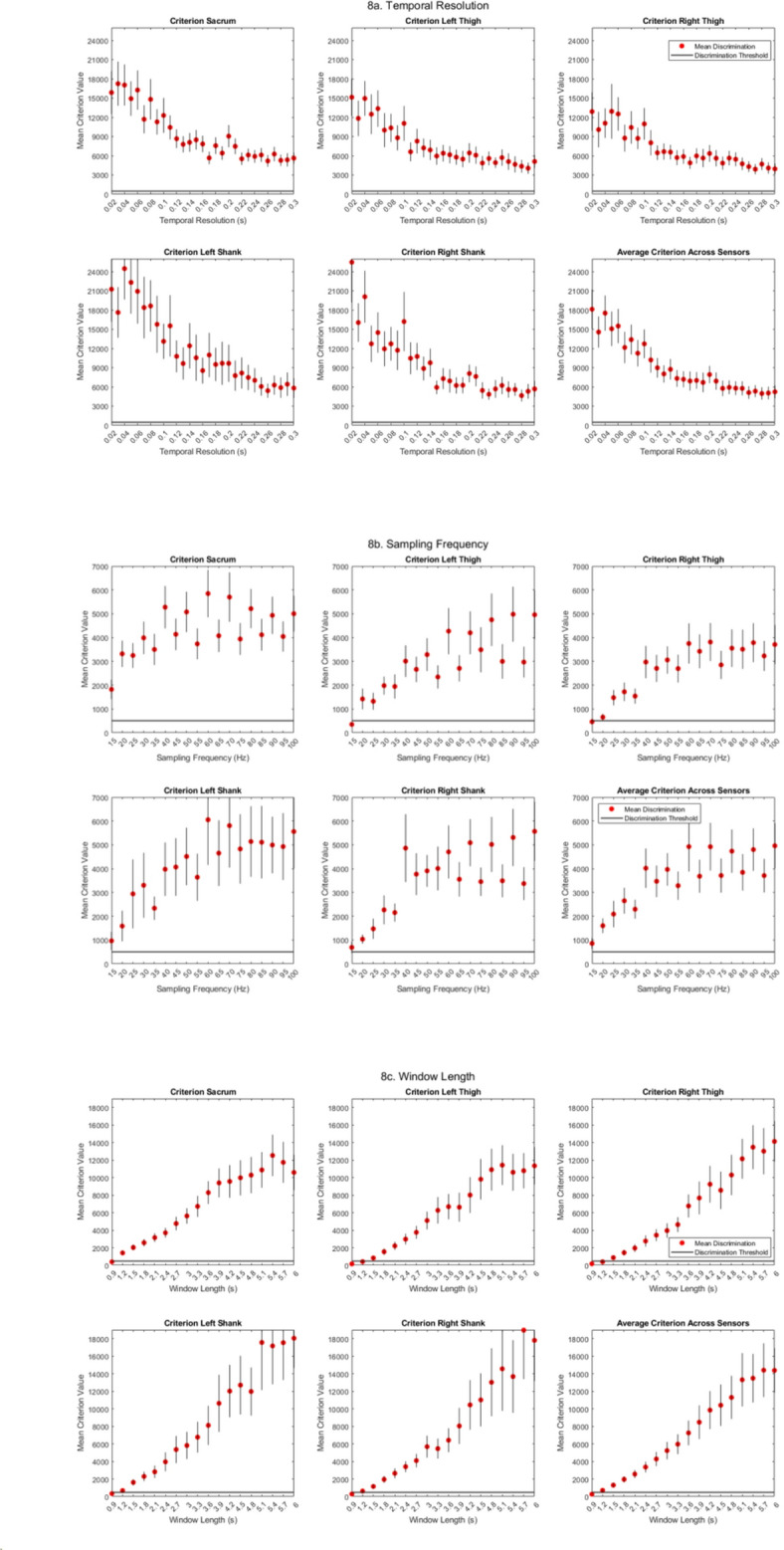

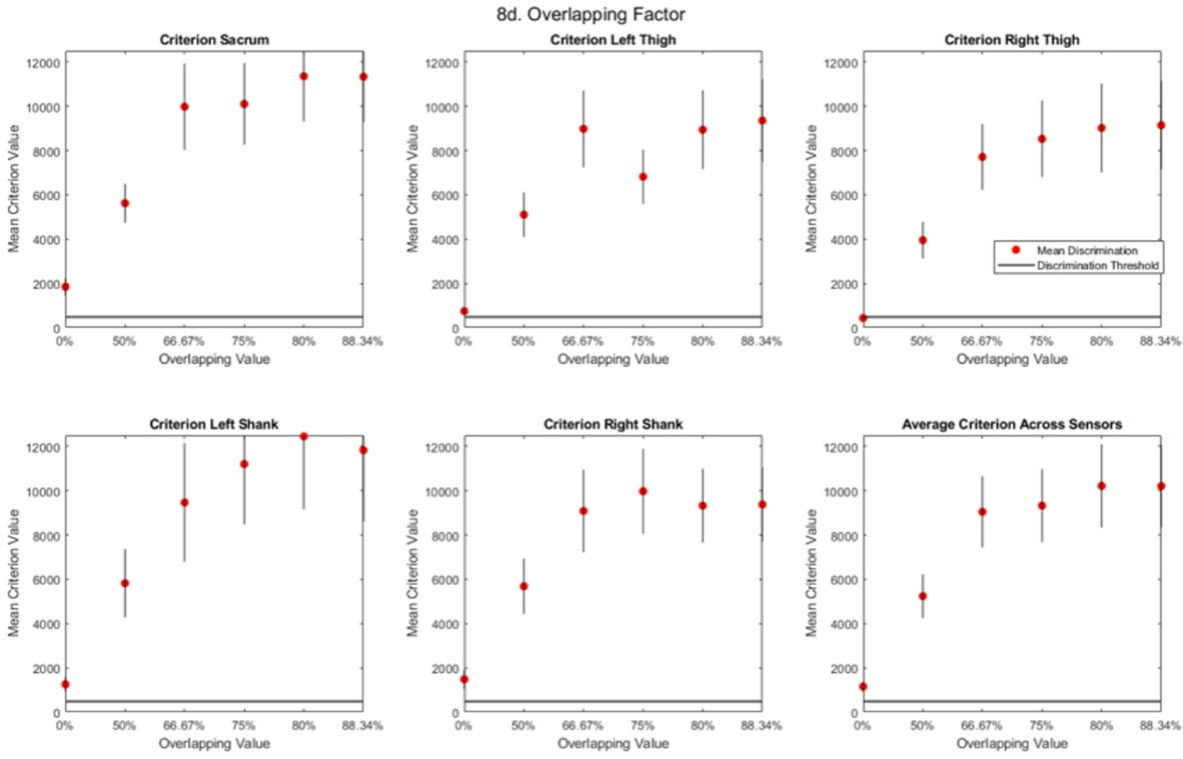
All participant (mean +/- standard error) criterion scores for each iteration for the (**a**) temporal resolution parameter at all five sensor locations, (**b**) the sampling frequency parameter at all five sensor locations, (**c**) the window-length parameter at all five sensor locations, and (**d**) the overlapping value parameter at all five sensor locations.

The optimal temporal resolution (Fig. 8a) for all five sensors was found to be 20 ms, with the highest resolution of 300 ms resulting in the lowest (therefore the least successful discrimination) criterion value. At the lowest temporal resolution however, the sensors with the highest mean criterion scores changed to the shanks (mean criterion of left and right shank equals 24942), followed by the sacrum (mean criterion equals 17248) followed by the thighs (mean criterion equals 14031). Occasional spikes in the curves between temporal resolutions can be accounted for by noise as each run comprises its own numerical search. The primary trend, however, is that a lower temporal resolution equates to a higher criterion value and, therefore, more successful discrimination. However, note that all of the temporal resolutions resulted in a mean score that resulted in a successful outcome.

### Sampling frequency

Increasing the sampling frequency (Fig. 8b) resulted in higher criterion values for classification, resulting in the same outcome as the baseline for a sampling frequency of 100 Hz. However, from a frequency of 40 Hz, the improvement in criterion appeared to plateau across the sensors.

### Window length

Window length explorations notable changes in criterion score (Fig. 8c). As the length of the window increased, the criterion score increased. A window length below 2 s resulted in a mean score that unsuccessfully discriminates locomotor activities. Longer window lengths changed the order of the most successful sensors, with the highest window length seeing the shanks changing to be the most successful sensor location (mean criterion equalling 18510), followed by the thighs (mean criterion equalling 12772), followed by the sacrum (mean criterion equalling 12523).

### Overlapping value

An overlapping value of 66% and over showed the highest mean criterion score across all sensors (Fig. 8d). The trend showed the largest increase in criterion scores for all sensors from an overlap factor of 0%-66%, with the sacrum remaining as the most successful location at higher overlapping factors (mean criterion of 11336), followed by the shanks (mean criterion of 10604), and then the thighs (mean criterion of 9251).

## Discussion

The current study explored the optimisation for sampling frequency, window length, temporal resolution, overlapping value and normalisation in a feature selection-based machine learning algorithm to best identify three locomotion activities from wearable sensors at different attachment locations. The study showed that when applying a machine learning-based algorithm using Principal Component Analysis and Discriminant Function Analysis to a Two-Dimensional Fourier Transform to discriminate between activities based on raw acceleration signals, non-normalised signals with a low temporal resolution (0.02–0.06 s), a mid to high ranged sampling frequency (40 –100 Hz), high window-length longer than two seconds, and a mid to high ranged overlapping value (50% − 88.34%) yielded the best discrimination. The study also found that manipulating parameter settings can influence which attachment location yields the highest discrimination accuracy for locomotor activities. When using optimal (longer) window lengths, the shanks demonstrated greater discrimination ability than the sacrum compared to baseline results. Similarly, with optimal temporal resolutions (shorter), the shanks emerged as the best-performing location, followed by the thighs and then the sacrum.

While the primary aim of this study was to optimise feature-based parameters for machine learning algorithms, the findings also have practical relevance for developers and end-users, such as clinicians and researchers. For instance, the results suggest that increasing window length enhances discrimination accuracy, with the greatest enhancements being seen in sensors attached to the shanks – which surpassed the discrimination ability of the sacrum at longer lengths. Importantly, even these longer window lengths remain relatively short and feasible for real-time clinical applications, where rapid detection of activities is essential for timely interventions, feedback, or monitoring. Shorter window lengths may offer faster detection but can risk reducing discrimination accuracy. Therefore, the choice of window length must balance the need for rapid identification with the model’s ability to accurately classify activities. Our parameter scans showed that window lengths of 1.8 s in all five sensor locations yielded a mean criterion score above the success threshold. Considering healthy individuals have a cadence of roughly two steps^30^ per second a window length of around 2 s allows for a minimum of four steps to be analysed within one feature window. For individuals with pathologies, such as lower limb amputations, who often have a lower cadence^31^, the window length may need to be adjusted to adequately capture enough steps in the gait cycle. This short window selection supports the findings of similar studies, which suggest that window lengths of 1–2 s are adequate for identifying activities of daily living^32,33^. Notably, selecting a window length of 1.8 s still produces a mean criterion score greater than 2000 across the five sensor locations. However, shorter windows introduce a risk of unsuccessful or miscategorisation when discriminating between cyclical gait modalities, with the mean criterion exhibiting unsuccessful discrimination for window lengths below 1.5 s in thigh-mounted sensors and 1.2 s in the sacrum and shanks.

The findings also indicate that a window overlapping value of 66% (or higher) yields the most favourable outcomes regarding discrimination criteria; beyond this point, the outcome across different sensors reaches a plateau. However, the current results also agree with studies suggesting that an overlapping window of 50%^26,34,35^ is adequate for achieving satisfactory classification outcomes, but 66% is optimal.

Regarding the signal sampling frequency, a frequency of 15 Hz was selected in the analysis as the lowest sampling frequency to analyse, as this would encompass the frequency of the all cyclical locomotor activities that clinicians or researchers may want to explore. Our results showed that a minimum sampling frequency of 20 Hz could successfully discriminate between experimental conditions in all five sensor locations, as a sampling rate of 15 Hz resulted in an unsuccessful criterion for the thigh. However, it should be considered that a frequency of 20 Hz bordered on the threshold required for successful discrimination. At 40 Hz the mean criterion score of the discrimination plateaued, with any variation in results over 40 Hz relating to noise. Previous research articles^10,12,19,20^ have used varying sampling frequencies with varying success. It has also been suggested that 99% of amplitude information from the Fast Fourier Transform spectrum resides within signal frequencies below 15 Hz for walking and 18 Hz for running^36,37^. This observation leads to the hypothesis that using sampling frequencies below 15 Hz could result in a loss of critical information, deteriorating the quality of discrimination. According to the Nyquist theorem, sampling frequencies should be at least twice the highest relevant signal frequency^38^; therefore, a minimum sampling rate of 30 Hz would be necessary to accurately capture the key motion frequencies for walking. However, this may not be sufficient when considering higher-frequency components, such as those associated with the foot striking the ground. If the waveform generated by foot impact provides valuable discriminatory information, a higher sampling rate would be required to capture these details accurately, as they occur at frequencies beyond the typical gait cycle speed. The present study, however, aimed to find optimal sampling frequencies. It was found that in order to assess gait for longer periods of time and/or discriminate between cyclical locomotor tasks, 40 Hz is the ideal sampling frequency. This frequency allows for maximum success of the machine learning algorithm while enabling users to preserve battery life by avoiding unnecessary higher rates. The ability to use lower power consumption facilitates longer recordings, which is particularly advantageous for continuous monitoring in real-world environments, such as tracking rehabilitation progress or assessing locomotor activity over extended periods. Additionally, using a 40 Hz sampling rate reduces the volume of data collected, easing computational and storage demands, which is crucial for resource-constrained applications or when analysing large datasets.

A key consideration in processing acceleration signals is whether to normalise amplitudes, as this step can influence the success of machine learning algorithms in classifying gait modalities. While more than half of studies in this field choose to normalise signals^28^the current study found that normalisation reduced the algorithm’s success rate. This suggests that variations in acceleration magnitudes, such as those arising from walking speed – whereby an individual walking quickly would typically strike the floor more forcefully compared to when walking slowly, provide meaningful information for discriminating between gait modalities.

The lowest temporal resolution was shown to provide the best outcome for discriminating experimental conditions. This suggests that the spectral repetition of the 2DFT is more important for discrimination than the nature of the accelerometer spikes in waveform. The spectrum’s details reveal the nature of the individual’s acceleration waveform, which appears to be less of a defining factor than the waveforms repeatability in terms of gait modality recognition. However, it is worth noting that the algorithm demonstrated success at all temporal resolutions explored, suggesting that features in the spectrum amplitude frequencies also provide valuable, discriminatory information. Our findings indicate that while spectral repetition is a dominant factor in discriminating gait modalities, the waveform’s shape and amplitude fluctuations also provide valuable information. For example, changes in acceleration spikes associated with foot strike and push-off phases contribute to the unique frequency patterns captured by each sensor. These shape-based features, in addition to repetitive spectral characteristics, enhance the algorithm’s ability to distinguish between subtle variations in gait modalities. Strictly speaking the data from Fig. 8a may be seen (particularly when averaged, as seen in the last subplot) to exhibit two optima, at 0.02 s and 0.2 s. This would further substantiate the relevance of the spectral shape in the success of the discrimination, although more data is required to improve the smoothness of the curve, and confirm whether these two peaks are indeed meaningful. The extent of the error bars at low values of temporal resolution clearly allow the possibility for non-minimal optimum value for this parameter. Finally, the optimisation of the machine learning was done multiple times, on data originating from one particular individual, and there are many specific individuals for which clear optimum temporal resolution is identified which is not the minimum value.

Note that the current algorithm was trained for each individual, with results indicating that not normalising signal amplitudes improved discrimination between locomotor activities. If the model were instead global, trained across all participants, the effects of normalisation might differ, as inter-participant variability could influence model performance^39^. Clinicians and researchers must consider the specific use case when deciding whether to normalise accelerometer data. While normalisation reduces variability introduced by factors such as slight changes in sensor placement (e.g., a slightly more proximal or distal location on the shank), footwear differences, and varying ground surfaces, it is important to acknowledge its trade-offs. It is logical that normalisation may weaken the output, as signal amplitude often holds valuable discriminatory information. However, normalisation is a necessary compromise; the slight deterioration in discrimination due to normalisation is acceptable and often worthwhile, as it enhances the model’s resilience to variations across participants, sensor placements, and experimental conditions. In this study, we are satisfied with the decision to normalise and the resulting model performance, as it successfully addressed inter-participant variability and other practical challenges.

Although this study found uniform parameter optimisation across all sensor locations, it was shown that certain parameters improved discrimination at locations with greater success than others, as different locations may capture unique aspects of gait dynamics. For example, sensors at the shanks experience larger amplitude movements than those at the sacrum, which may have influenced the effectiveness of parameters, such as window length or temporal resolution, which saw the shanks becoming the most successful attachment locations for the discrimination of the locomotor activities. In studies involving pathological gait, such as individuals with amputation or reduced mobility, location-specific parameter adjustments may improve discrimination by accounting for unique movement characteristics at each sensor site. Additionally, this study underscores the practical relevance of choosing appropriate parameters in wearable gait analysis. For clinicians and researchers, understanding the interplay between waveform shape and spectral repetition could aid in tailoring algorithms for specific gait analyses. This insight is particularly valuable when sensors are used in variable environments or with individuals whose gait may change over time.

### Limitations

One limitation of the current study is the number of activities used. We assessed three separate walking speeds as a method to test the sensitivity and specificity of our algorithm. Future works may look at adding additional cyclical gait modalities, such as stair negotiation or slope ascent and descent, to assess whether the parameter settings align with this study. Additionally, we utilised a model tailored to each sensor in each individual. Future studies wishing to explore a model with all individual data trained together globally may find differences in our results.

## Conclusion

This study systematically optimised feature-based parameters for feature-based machine learning algorithms to classify locomotor tasks using wearable sensors, with consistent results across different sensor attachment locations. Non-normalised signals, low temporal resolutions (0.02–0.06 s), a sampling frequency of 40 Hz, a window length of 2 s, and an overlapping value of 66% were identified as optimal for accurate gait classification. Additionally, longer window lengths and shorter temporal resolutions changed the most successful sensor attachment location from that of the sacrum, to those of the shanks. While spectral repetition was a dominant discriminating factor, waveform shape and amplitude fluctuations also contributed valuable information. These findings provide practical guidance for tailoring wearable sensor systems to enhance activity recognition in diverse applications.

## Data Availability

The datasets generated and/or analysed during the current study are available in the following github.com repository: https://github.com/Liamdhughes/activity_discrimination_PCA_DFA.

## References

[1] Li, Z. et al. An adaptive hidden Markov model for activity recognition based on a wearable multi-sensor device. *J. Med. Syst.* **39**, 57 (2015). 25787786 10.1007/s10916-015-0239-x PMC5729042

[2] Beaufils, B., Chazal, F., Grelet, M. & Michel, B. Robust stride detector from ankle-mounted inertial sensors for pedestrian navigation and activity recognition with machine learning approaches. *Sensors* **19**, 4491 (2019). 31623248 10.3390/s19204491 PMC6833053

[3] Mahoney, J. M. & Rhudy, M. B. Methodology and validation for identifying gait type using machine learning on IMU data. *J. Med. Eng. Technol.* **43**, 25–32 (2019). 31037995 10.1080/03091902.2019.1599073

[4] Hu, B., Dixon, P. C., Jacobs, J. V., Dennerlein, J. T. & Schiffman, J. M. Machine learning algorithms based on signals from a single wearable inertial sensor can detect surface- and age-related differences in walking. *J. Biomech.* **71**, 37–42 (2018). 29452755 10.1016/j.jbiomech.2018.01.005

[5] Mannini, A. & Sabatini, A. M. Accelerometry-based classification of human activities using Markov modeling. *Comput. Intell. Neurosci.* **2011**, 1–10 (2011). 10.1155/2011/647858 PMC3166724 21904542

[6] Aziz, O., Musngi, M., Park, E. J., Mori, G. & Robinovitch, S. N. A comparison of accuracy of fall detection algorithms (threshold-based vs. machine learning) using waist-mounted tri-axial accelerometer signals from a comprehensive set of falls and non-fall trials. *Med. Biol. Eng. Comput.* **55**, 45–55 (2017). 27106749 10.1007/s11517-016-1504-y

[7] Daines, K. J. F., Baddour, N., Burger, H., Bavec, A. & Lemaire, E. D. Fall risk classification for people with lower extremity amputations using random forests and smartphone sensor features from a 6-minute walk test. *PLoS ONE*. **16**, e0247574 (2021). 33901209 10.1371/journal.pone.0247574 PMC8075234

[8] Altilio, R., Rossetti, A., Fang, Q., Gu, X. & Panella, M. A comparison of machine learning classifiers for smartphone-based gait analysis. *Med. Biol. Eng. Comput.* **59**, 535–546 (2021). 33548017 10.1007/s11517-020-02295-6 PMC7925506

[9] Kouris, I. & Koutsouris, D. A comparative study of pattern recognition classifiers to predict physical activities using smartphones and wearable body sensors. *THC* **20**, 263–275 (2007). 10.3233/THC-2012-0674 23000559

[10] Soltani, A., Dejnabadi, H., Savary, M. & Aminian, K. Real-World gait speed estimation using wrist sensor: A personalized approach. *IEEE J. Biomed. Health Inf.* **24**, 658–668 (2020). 10.1109/JBHI.2019.2914940 31059461

[11] Dixon, P. C. et al. Machine learning algorithms can classify outdoor terrain types during running using accelerometry data. *Gait Posture*. **74**, 176–181 (2019). 31539798 10.1016/j.gaitpost.2019.09.005

[12] Chen, W. H. et al. Determining motions with an IMU during level walking and slope and stair walking. *J. Sports Sci.* **38**, 62–69 (2020). 31623527 10.1080/02640414.2019.1680083

[13] Chomiak, T., Xian, W., Pei, Z. & Hu, B. A novel single-sensor-based method for the detection of gait-cycle breakdown and freezing of gait in parkinson’s disease. *J. Neural Transm*. **126**, 1029–1036 (2019). 31154512 10.1007/s00702-019-02020-0

[14] Rehman, R. Z. U. et al. Comparison of walking protocols and gait assessment systems for machine learning-based classification of parkinson’s disease. *Sensors* **19**, 5363 (2019). 31817393 10.3390/s19245363 PMC6960714

[15] Goodlich, B. I. et al. Machine learning to quantify habitual physical activity in children with cerebral palsy. *Dev. Med. Child. Neurol.* **62**, 1054–1060 (2020). 32420632 10.1111/dmcn.14560

[16] Trentzsch, K. et al. Using machine learning algorithms for identifying gait parameters suitable to evaluate subtle changes in gait in people with multiple sclerosis. *Brain Sci.* **11**, 1049 (2021). 34439668 10.3390/brainsci11081049 PMC8391565

[17] Mellema, M. & Gjøvaag, T. Reported outcome measures in studies of real-world ambulation in people with a lower limb amputation: A scoping review. *Sensors* **22**, 2243 (2022). 35336412 10.3390/s22062243 PMC8955603

[18] Hughes, L. D., Bencsik, M., Bisele, M. & Barnett, C. T. Using lower limb wearable sensors to identify gait modalities: A machine-learning-based approach. *Sensors* **23**, 9241 (2023). 38005627 10.3390/s23229241 PMC10675053

[19] Lee, M. W., Khan, A. M. & Kim, J. H. Young-Sun Cho, & Tae-Seong Kim. A single tri-axial accelerometer-based real-time personal life log system capable of activity classification and exercise information generation. In *Annual International Conference of the IEEE Engineering in Medicine and Biology* 1390–1393. 10.1109/IEMBS.2010.5626729 (IEEE, 2010). 10.1109/IEMBS.2010.5626729 21096339

[20] Noh, B. et al. Prediction of decline in global cognitive function using machine learning with feature ranking of gait and physical fitness outcomes in older adults. *IJERPH* **18**, 11347 (2021). 34769864 10.3390/ijerph182111347 PMC8582857

[21] Xu, H., Liu, J., Hu, H. & Zhang, Y. Wearable sensor-based human activity recognition method with multi-features extracted from Hilbert-Huang transform. *Sensors* **16**, 2048 (2016). 27918414 10.3390/s16122048 PMC5191029

[22] Anh, N. X., Nataraja, R. M. & Chauhan, S. Towards near real-time assessment of surgical skills: A comparison of feature extraction techniques. *Comput. Methods Programs Biomed.* **187**, 105234 (2020). 31794913 10.1016/j.cmpb.2019.105234

[23] Ramsey, M. T. The ethology of honeybees (Apis mellifera) studied using accelerometer technology. (2018).

[24] Bax, A. & Lerner, L. Two-dimensional nuclear magnetic resonance spectroscopy. *Science* **232**, 960–967 (1986). 3518060 10.1126/science.3518060

[25] Griffiths, B., Diment, L. & Granat, M. H. A machine learning classification model for monitoring the daily physical behaviour of lower-limb amputees. *Sensors* **21**, 7458 (2021). 34833534 10.3390/s21227458 PMC8625063

[26] Janidarmian, M., Roshan Fekr, A., Radecka, K. & Zilic, Z. A comprehensive analysis on wearable acceleration sensors in human activity recognition. *Sensors* **17**, 529 (2017). 28272362 10.3390/s17030529 PMC5375815

[27] Farrahi, V. Calibration and validation of accelerometer-based activity monitors_ A systematic review of machine-learning approaches. *feature extraction* (2019). 10.1016/j.gaitpost.2018.12.003 30579037

[28] Figueiredo, J., Santos, C. P. & Moreno, J. C. Automatic recognition of gait patterns in human motor disorders using machine learning: A review. *Med. Eng. Phys.* **53**, 1–12 (2018). 29373231 10.1016/j.medengphy.2017.12.006

[29] Bisele, M., Bencsik, M., Lewis, M. G. C. & Barnett, C. T. Optimisation of a machine learning algorithm in human locomotion using principal component and discriminant function analyses. *PLoS ONE*. **12**, e0183990 (2017). 28886059 10.1371/journal.pone.0183990 PMC5590884

[30] Tudor-Locke, C. et al. How fast is fast enough? Walking cadence (steps/min) as a practical estimate of intensity in adults: a narrative review. *Br. J. Sports Med.* **52**, 776–788 (2018). 29858465 10.1136/bjsports-2017-097628 PMC6029645

[31] Waters, R., Perry, J., Antonelli, D. & Hislop, H. Energy cost of walking of amputees: the influence of level of amputation. (1976). 1249111

[32] Fida, B., Bernabucci, I., Bibbo, D., Conforto, S. & Schmid, M. Varying behavior of different window sizes on the classification of static and dynamic physical activities from a single accelerometer. *Med. Eng. Phys.* **37**, 705–711 (2015). 25983067 10.1016/j.medengphy.2015.04.005

[33] Bersch, S., Azzi, D., Khusainov, R., Achumba, I. & Ries, J. Sensor data acquisition and processing parameters for human activity classification. *Sensors* **14**, 4239–4270 (2014). 24599189 10.3390/s140304239 PMC4003942

[34] Cleland, I. et al. Optimal placement of accelerometers for the detection of everyday activities. *Sensors* **13**, 9183–9200 (2013). 23867744 10.3390/s130709183 PMC3758644

[35] Bao, L. & Intille, S. S. Activity recognition from user-annotated acceleration data. In *Pervasive Computing* (eds Ferscha, A. & Mattern, F.) vol 3001, 1–17 (Springer Berlin Heidelberg, 2004).

[36] Antonsson, E. K. & Mann, R. W. The frequency content of gait. *J. Biomech.* **18**, 39–47 (1985). 3980487 10.1016/0021-9290(85)90043-0

[37] Bhattacharya, A., McCutcheon, E. P., Shvartz, E. & Greenleaf, J. E. Body acceleration distribution and O2 uptake in humans during running and jumping. *J. Appl. Physiol.* **49**, 881–887 (1980). 7429911 10.1152/jappl.1980.49.5.881

[38] Austerlitz, H. CHAPTER 4 - Analog/digital conversions. In *Data Acquisition Techniques Using PCs (Second Edition)* (ed. Austerlitz, H.) 51–77 10.1016/B978-012068377-2/50004-8 (Academic Press, 2003).

[39] Jamieson, A., Murray, L., Stankovic, V., Stankovic, L. & Buis, A. Unsupervised cluster analysis of walking activity data for healthy individuals and individuals with lower limb amputation. *Sensors* **23**, 8164 (2023). 37836994 10.3390/s23198164 PMC10575014

